# Feasibility of improving cone‐beam CT number consistency using a scatter correction algorithm

**DOI:** 10.1120/jacmp.v14i6.4346

**Published:** 2013-11-04

**Authors:** Jun Li, Weiguang Yao, Ying Xiao, Yan Yu

**Affiliations:** ^1^ Department of Radiation Oncology Thomas Jefferson University Philadelphia PA USA; ^2^ Department of Medical Physics Eastern Health ‐ Cancer Care Program St. John's Newfoundland Canada

**Keywords:** cone‐beam computed tomography, cone‐beam CT number, scatter correction

## Abstract

The study was to explore the feasibility of improving cone‐beam CT (CBCT) number (corresponding to the Hounsfield units in computed tomography) consistency using a scatter‐correction algorithm, with the aim of using CBCT images for treatment planning with density correction. A scatter‐correction algorithm was applied to a Varian OBI CBCT and an Elekta XVI CBCT, and was evaluated for improving CBCT number consistency. CBCT numbers of phantom materials were compared between images with and without bolus, which introduced additional scatter, and with and without scatter‐correction processing. It was observed that CBCT numbers were different in the images with and without bolus in both CBCT studies, and the differences were reduced remarkably after scatter‐correction processing. Results showed that CBCT number consistency was significantly improved by use of the scatter‐correction algorithm.

PACS number: 87.55‐x

## I. INTRODUCTION

In radiation therapy, cone‐beam computed tomography (CBCT) has been used to verify patient positioning and target localization before treatment. Studies applying CBCT images for treatment planning have been reported,^1^, ^2^, ^3^, ^4^, ^5^, ^6^, ^7^, ^8^ which were aimed to perform replanning using cone‐beam images when changes in the patient anatomy are detected before a treatment. Real‐time replanning will allow modification of the initial treatment plan to accommodate the variations while the patient is on the treatment couch. To perform density‐corrected treatment planning, CBCT number (corresponding to the Hounsfield units (HU) in computed tomography CT) to electron density (ED) conversion needs to be applied. Scatter is a major issue in CBCT, which affects image quality and CBCT number. It has been reported that scatter caused decreased image quality and incorrect HU (i.e., CBCT number) in CBCT.^(3)^ It was found that unlike conventional computed tomography, where CT‐to‐ED conversion was consistent, CBCT number‐to‐ED conversion in CBCT changed under different volume scanning.^(9)^ Several methods have been studied for using CBCT images for treatment planning, which include: correlating CBCT number and CT number of regions of interest to create CBCT‐density conversion;^(^ ^6^ ^,^ ^10^ ^)^ employing scatter‐correction methods to improve CBCT number accuracy;^11^, ^12^, ^13^ and using image registration of CT and CBCT to map CT number to CBCT.^(^ ^7^ ^,^ ^14^ ^)^

The study here was to evaluate a scatter‐correction method^(^ ^15^ ^,^ ^16^ ^)^ for improving CBCT number consistency. There have been many scatter‐correction methods developed to improve CBCT image quality.^(17)^ The scatter‐correction method used in this study was developed recently for CBCT, and an experimental study on a Siemens MV CBCT has demonstrated improvement of the accuracy of linear attenuation coefficients by use of the scatter‐correction method.^(16)^ In this study, we applied the scatter‐correction method to kV CBCT and examined its performance on a Varian Trilogy OBI CBCT and an Elekta XVI CBCT for improving CBCT number consistency, with the ultimate goal of using CBCT images for treatment planning with density correction.

## II. MATERIALS AND METHODS

Experiments were conducted at two institutions on a Varian OBI CBCT (Varian Medical Systems, Palo Alto, CA) and an Elekta XVI CBCT (Elekta, Stockholm, Sweden), respectively. The study was aimed to test the scatter‐correction algorithm on two imaging systems and was not aimed to compare the experiments between the systems. Each institution used available phantoms in the study.

### A. Phantom and image acquisition

In the study of Varian OBI CBCT, a Catphan phantom (The Phantom Laboratory Inc., Salem, NY) was scanned with and without a 3 cm bolus with a pelvis protocol (125 kVp, 80 mA with a full bowtie filter and full range scan). The 3 cm bolus was made of a few pieces of 1 cm thick bolus, which was used to introduce additional scatter and to test the robustness of the scatter correction algorithm on variant phantom or patient body. The CBCT numbers of phantom inserts on the images with and without the scatter correction were checked.

In the study of Elekta XVI CBCT, a Gammex tissue characterization phantom (Gammex 467; Gammex Inc., Middleton, WI) was used to examine CBCT number consistency, which was scanned with and without a bolus. The phantom, which is popularly used in clinics for creating CT‐ED conversion for treatment planning, has 16 insert materials with different densities varying from low density (e.g., lung density) to high density (e.g., cortical bone density). CBCT numbers of the insert materials were measured on the CBCT images. Further, an anthropomorphic pelvis RANDO phantom (The Phantom Laboratory) was used to validate the scatter correction by evaluating dose calculation on the CBCT images of the phantom. A clinical prostate protocol was used in these CBCT scans (120 KVp, 40 mA, with a full range scan). Bowtie filters were not used in the scans unless it is particularly mentioned.

### B. Scatter correction

An algorithm based on the scatter‐correction method^(^ ^15^ ^,^ ^16^ ^)^ was developed to process CBCT projection images. In the scatter‐correction algorithm, the first order scatter fluence, $S_{1}$, was expressed as a function of the primary photon fluence, P, and the higher order scatter fluence, $S_{h}$, was approximated to be either a constant, b, or proportional to the first order scatter fluence, ${\text{aS}}_{1}$, where a and b are parameters. Namely, a CBCT projection, I, was approximately expressed by $I=P+S_{1}(P)+b\;\text{or}\;I=P+(1+a)S_{1}(P)$. The form of $S_{1}(P)$ was derived based on the Klein‐Nishina formula.^(15)^ An iterative approach $P^{0}=I,\;P^{k+1}=P^{k}+c\;(I-P^{k}-S_{1}-S_{h}),\;k=1,\;2,\;\ldots$, was used to estimate primary and scatter fluences from the projections. Here c is another parameter and was taken as unity in the iteration. More details about the iteration can be found in the published study.^(15)^ The iteration converged rapidly, only three iterations were sufficient to reach a stable projection P.

For the Varian OBI system, antiscatter grids are employed before the photons arrive at the detector. During CBCT reconstruction, no further scatter correction was performed. Our scatter‐correction algorithm adopted $S_{h}={\text{aS}}_{1}$ to do the scatter correction. The functions of bowtie filter and antiscatter grid were modeled in the algorithm. To determine the value of a, BEAMnrc Monte Carlo simulation software^(18)^ was used to generate the primary projection and whole (primary plus scatter) projection of a cylindrical water phantom. The value was then obtained by using our scatter‐correction algorithm when the difference was minimal between the estimated primary fluence from the whole projection and the true primary projection. The Elekta XVI system uses a scatter‐correction scheme called “uniform scatter correction”. We found that it was better to further reduce scatter by applying our scatter correction, letting $S_{h}=b$, where b was optimally determined for each projection by searching the value b so that $I-P_{k}-S_{1}-S_{h}$ was minimized.

### C. Evaluation of CBCT number consistency

Because Varian OBI system does not allow feeding back the processed projections into the acquisition‐reconstruction loop, an in‐house algorithm based on algebraic reconstruction technique (ART)^(19)^ was used to perform the reconstruction. To evaluate the effect of scatter‐correction processing, the CBCT numbers of the inserts reconstructed from the scatter‐corrected projections were compared with those from the original projections.

In the study of Elekta XVI CBCT, the processed projection images were imported into the CBCT systems and volumetric images were reconstructed. CBCT numbers of the materials of the Gammex phantom were measured on the XVI and were compared with those of the images without scatter correction. A test based on the Gammex phantom was conducted to evaluate density‐corrected dose calculations using the CBCT‐to‐ED conversions generated from the images with and without scatter correction, respectively. Two CBCT‐to‐ED conversion files were generated from the nonbolused images (one set of images was processed with scatter‐correction and the other one was not). The CBCT‐to‐ED conversion generated from processed nonbolused images was applied to the processed bolused images, and the conversion generated from nonprocessed nonbolused images was applied to the nonprocessed bolused images. Single field treatment plans were generated in a CMS XiO treatment planning system (CMS Inc., St. Louis, MO), where an anterior beam of 6 MV with field size of $2\times 10\;{\text{cm}}^{2}$ was applied to the Gammex phantom. The dose calculations were compared with a plan using CT images and clinical CT‐to‐ED conversion. The latter was used as the standard.

An experiment using the anthropomorphic pelvis RANDO phantom was conducted to further evaluate the scatter correction in the XVI CBCT. Four‐field pelvis treatment plans were generated using the CBCT images and CT images of the RANDO phantom, respectively. The size of each treatment field was $9\times 9\;{\text{cm}}^{2}$. Same beam arrangement and monitor units were applied to the CBCT plans which were based on the original CBCT images and the scatter‐correction processed images, respectively, and the CT plan. The CBCT‐to‐ED conversion generated from scatter‐correction processed images of the Gammex phantom was applied to the processed RANDO phantom images, and the conversion generated from nonprocessed images was applied to the nonprocessed RANDO phantom images. Clinical CT‐ED conversion was used in the CT plan dose calculation. The point doses and dose‐volume histograms (DVHs) were compared to check the effect of scatter correction on dose calculation.

## III. RESULTS & DISCUSSION

**Figure 1:**
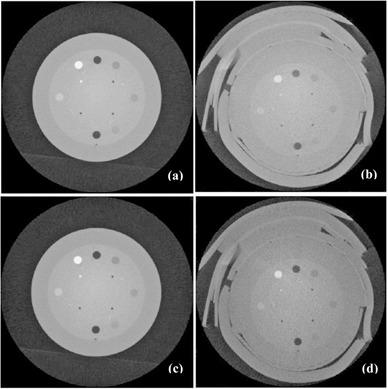
Results of Varian OBI CBCT. Images of a Catphan phantom (a) without bolus, without scatter‐correction processing; (b) with a 3 cm bolus, without scatter‐correction processing; (c) without bolus, with scatter‐correction processing; (d) with a 3 cm bolus, with scatter‐correction processing.

Figure 1 demonstrates the effect of scatter correction on image quality on the Varian CBCT images. Figures 1(a) and 1(b) are images reconstructed from the original projections, without bolus and with bolus, respectively. Figures 1(c) and 1(d) are images reconstructed from scatter corrected projections. Image contrast‐to‐noise ratio (CNR)^(20)^ was improved with the scatter correction; the CNRs were 0.6–3.8 in the original images and 1.3–4.0 in the scatter corrected images, with average increase of 0.8.

**Figure 2:**
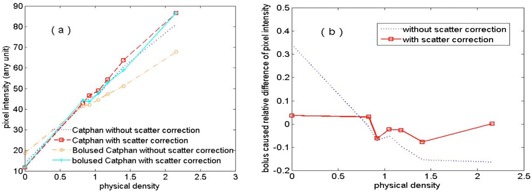
Results of Varian OBI CBCT: (a) image pixel intensity vs. material physical density; (b) relative difference of image pixel intensity vs. material physical density.

Figure 2 demonstrates the effect of scatter correction on CBCT number consistency. Figure 2(a) shows image pixel intensity (proportional to CBCT number) of the inserts of Catphan phantom, vs. material physical density, for bolused, nonbolused, scatter‐corrected, and noncorrected cases, respectively. Figure 2(b) shows pixel intensity differences of the inserts in the corresponding nonbolused and bolused images, for scatter corrected and noncorrected cases.

It is shown from Figs. 2(a) and 2(b) that the application of the scatter correction significantly improved the consistency of pixel intensity (i.e., the consistency of CBCT number). Without scatter correction, the added bolus caused 34% and 16% changes of pixel intensities of the air and Teflon, respectively. While with scatter correction, the changes were within 7% for all inserts. The consistency is desired in CBCT‐based dose calculation and can greatly increase the dose calculation accuracy after performing transformation from CBCT numbers to the CT numbers of the inserts.^(6)^

**Figure 3:**
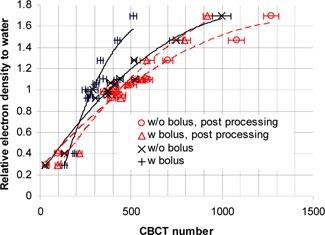
Results of Elekta XVI CBCT: relative electron density vs. measured CBCT numbers. The symbols represent the data points and the lines represent the trendlines (the solid lines and dashed lines are for the data without and with scatter‐correction processing, respectively). A 1 cm bolus was used in the bolused case.

Figure 3 shows the CBCT‐to‐ED curves obtained on the Elekta XVI CBCT. The symbols represent the data points and the curves represent the trends of the variation of CBCT numbers with electron densities. The solid and dashed lines represent nonprocessed data and correction‐processed data, respectively. The curves for bolused case and nonbolused case, which are supposed to be the same (i.e., should overlay on each other), are quite different. The CBCT number inconsistency was caused by the effect of increased scatter‐primary ratio due to the bolus on CBCT image acquisition. More significant differences between bolused and nonbolused cases were observed in the results without the scatter‐correction processing. With scatter correction, the differences were greatly reduced. Our scatter correction improved the CBCT number consistency in the XVI system. However, compared to the performance of our scatter‐correction algorithm in the OBI system, the resulted CBCT number consistency in XVI was not as good as that in OBI. This probably was due to the independent scatter correction done by XVI. The extra scatter correction may also explain the streaking artifacts in the XVI reconstructed images.

Table 1 lists the CBCT numbers of each material of the Gammex phantom in bolused and nonbolused cases, with and without scatter‐correction processing, respectively. Without scatter correction, the mean difference between bolused and nonbolused cases among all the materials was 128 (minimum: 40; maximum: 485; standard deviation: 118). With scatter correction, the mean difference was reduced to 80 (minimum: 4; maximum: 349; standard deviation: 99).

Further, the CBCT numbers obtained from the Elekta CBCT images with scatter‐correction processing were compared with those obtained from the images acquired with a bowtie filter. With a bowtie filter, the differences of CBCT numbers between nonbolused and bolused conditions were reduced because the bowtie filter hardens the kV beam and thus decreases the scatter‐primary ratio of photon fluences at the detector. Table 2 lists the summary of the CBCT number differences of the Gammex phantom inserts between images with and without a 1 cm bolus, for the original images (no scatter‐correction processing, no bowtie filter), scatter‐correction processed image (no bowtie filter), and the images acquired with a bowtie filter. The differences of the CBCT numbers between bolused and nonbolused conditions in the scatter‐correction processed images were smaller than those in the images acquired with a bowtie filter.

**Figure 4:**
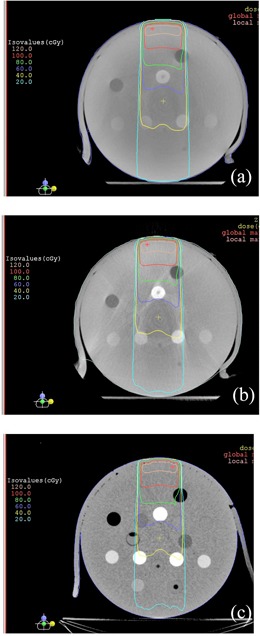
Comparison of isodose distributions of single beam bolused plans based on the Gammex phantom: (a) CBCT plan using images without scatter‐correction processing, (b) CBCT plan using images with scatter‐correction processing, and (c) CT plan. The CBCT images were acquired with an Elekta XVI CBCT.

Figure 4 shows comparison of dose calculations among the single field treatment plans using scatter‐corrected and noncorrected CBCT images of the Gammex phantom acquired from the Elekta XVI CBCT, and those from a CT. Compared to the CBCT plan using images without scatter correction, the CBCT plan using images with scatter correction shows that the dose distribution was closer to that in the CT plan (the standard). The dose difference at the marked isocenter was 7.1% between the CT plan and the CBCT plan using images without correction. The difference was reduced to 0.8% after the images were scatter corrected.

**Table 1:** CBCT numbers of various Gammex phantom inserts measured in images with/without 1 cm bolus, and before (“Original”) and after (“Processed”) the scatter‐correction processing. The images were acquired with an Elekta XVI CBCT

		*Original*		*Processed*	
*Insert Material*	*Electron Density to Water*	*Without Bolus*	*With Bolus*	*Without Bolus*	*With Bolus*
		CBCT±std	CBCT±std	CBCT±std	CBCT±std
LN‐lung 300	0.289	27±12	134±12	33±19	97±22
Lung 450	0.403	135±13	190±10	97±18	218±19
Adipose	0.924	305±16	258±10	397±26	441±21
Breast	0.956	376±24	275±13	438±31	394±27
Solid Water 1	0.989	435±22	334±14	509±30	515±26
Solid Water 2	0.989	438±12	377±13	430±15	426±19
Solid Water 3	0.989	372±14	264±11	403±25	349±24
Solid Water 4	0.989	360±12	320±11	432±14	423±24
Water	1	292±28	247±17	406±26	389±23
Brain	1.049	413±10	300±7	501±31	480±32
Liver	1.064	389±18	308±15	497±17	417±19
Inner Bone	1.096	523±19	421±17	594±25	563±19
B‐200	1.106	440±19	345±18	580±19	534±18
CB2 (30%)	1.279	516±15	330±14	700±27	588±30
CB2 (50%)	1.470	749±36	424±13	1078±45	793±43
Cortical Bone	1.695	996±51	511±19	1269±43	920±29

**Table 2:** Summary of CBCT number differences of the Gammex phantom inserts between images with and without 1 cm bolus. Results of the original images, scatter‐correction processed images, and images acquired with a bowtie filter are listed for comparison. All the images were acquired with an Elekta XVI CBCT

*CBCT Number Difference*	*Original Images*	*Scatter‐correction Processed Images*	*Images Acquired With a Bowtie Filter*
Maximum	485	349	357
Mean	128	80	100
Minimum	40	4	6

**Figure 5:**
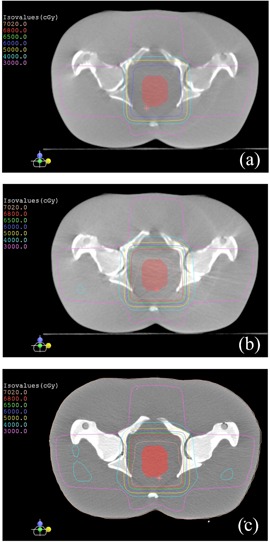
Comparison of isodose distributions of the four‐field treatment plans based on the pelvis RANDO phantom: (a) CBCT plan using images without scatter‐correction processing, (b) CBCT plan using images with scatter‐correction processing, and (c) CT plan. The CBCT images were acquired with an Elekta XVI CBCT.

**Figure 6:**
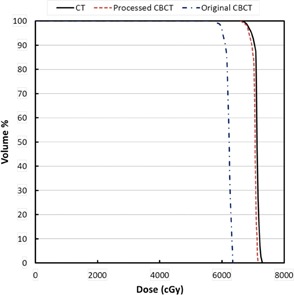
Comparison of target dose‐volume histograms among the four‐field treatment plans based on the pelvis RANDO phantom.

Figure 5 shows the results of the RANDO phantom study — comparison of dose distributions among the treatment plans using scatter‐corrected and noncorrected CBCT images acquired on the Elekta XVI CBCT, and CT images. With the same monitor units for each field, compared to the CT plan, the CBCT plan using original CBCT images (i.e., without scatter correction) had significantly different dose distribution (lower doses), and the dose distribution of the CBCT plan using scatter‐corrected images was close to that of the CT plan. The dose differences at isocenter which was in the target volume, were 11.4% and 0.8% between the CT plan and the noncorrected CBCT plan and the scatter‐corrected plan, respectively. Figure 6 shows the DVHs of the target. The target dose coverage of the CBCT plan using scatter‐corrected images was similar to that of the CT plan, whereas the DVH of the CBCT plan using noncorrected images was much inferior.

The results showed that by using the scatter‐correction processing, the CBCT number consistency was significantly improved on both Varian OBI CBCT and Elekta XVI CBCT. Although the Varian OBI CBCT system has a 10:1 antiscatter grid to reduce scatter, the residual scatter still significantly affects CBCT number consistency. In our study of the Catphan phantom with bolus, the maximum scatter‐primary ratio was about 60%. It is indicated that scatter correction is necessary for CBCT applications, especially for treatment planning that requires density correction.

Since it is software processing, scatter correction using the algorithm will have flexibility in the application to improve CBCT number consistency and image quality; scatter correction may be optimized under actual scanning conditions. When the scatter correction algorithm is applied together with a bowtie filter, scatter can be further reduced and the consistency of CBCT number can be improved.

The feasibility study has showed that it is promising to apply the scatter‐correction algorithm to improve CBCT number consistency for treatment planning. We are improving the algorithm and will report patient study in the future.

## IV. CONCLUSIONS

The preliminary study on the Varian OBI CBCT and Elekta XVI CBCT demonstrated that the CBCT number consistency was remarkably improved by using the scatter‐correction algorithm. We expect to conduct further study on the algorithm for additional improvement on the CBCT number consistency for clinical application.
